# The American Year-Book of Medicine and Surgery for 1902

**Published:** 1902-12

**Authors:** 


### Books and Magazines.

THE AMERICAN YEAR-BOOK OF MEDICINE AND SURGERY FOR 1902. A yearly digest of scientific progress and authoritative opinion in all branches of medicine and surgery, drawn from journals, monographs, and text-books of the leading American and foreign authors and investigators. Arranged, with critical editorial comments, by eminent American specialists, under the editorial charge of George M. Gould, A. M., M. D. In two volumes—Volume I, including General Medicine, octavo, 700 pages, illustrated; Volume II, General Surgery, octavo, 684 pages, illustrated. Philadelphia and London: W. B. Saunders & Co. 1902. Per volume: Cloth, $3.00 net; half Morocco, $3.75 net.

The plan of issuing the Year-Book in two volumes, inaugurated two years ago, met with such general favor with the profession that the publishers have decided to follow the same plan with all succeeding issues. Each volume is complete in itself, and the work is sold either separately or in sets.

The contents of these volumes, critically selected from leading journals, monographs, and text-books, is much more than a compilation of data. The extracts are carefully edited and commented upon by eminent specialists, the reader thus obtaining, not only a yearly digest of scientific progress and authoritative opinion in all branches of medicine and surgery, but also the invaluable annotations and criticisms of the editors, all leaders in their several specialties. As usual, this issue of the Year-Book is not lacking in its illustrative feature; for, besides a large number of text-cuts, the surgery volume contains five, and the medicine volume four, full-page inserts. In every way the Year-Book of 1902 fully upholds, if it does not strengthen, the reputation won by its predecessors.

Those in charge of the departments of Surgery are J. C. DaCosta, John H. Gibbon, B. C. Hirst, W. A. N. Dorland, J. M. Baldy, H. F. Hansell, W. Reber and C. H. Burnett, of Philadelphia; E. F. Ingalls, of Chicago; H. G. Ohls, of Odell, Ill.; C. A. Hamann, of Cleveland.

Of Medicine: A. Stengel, D. L. Edsall, L. Starr, Alf. Hand, Jr., D. Reisman, A. and J. Kelly, L. A. Duhring, M. B. Hartzell and A. A. Stevens, of Philadelphia; Arch. Church, of Chicago; R. W. Wilcox, of New York, etc.

